# Orbital metastasis as the primary presentation of nasopharyngeal carcinoma^☆^

**DOI:** 10.1016/j.bjorl.2015.04.006

**Published:** 2015-09-09

**Authors:** Sung-Chan Shin, Sung-Lyong Hong, Chang-Hoon Lee, Kyu-Sup Cho

**Affiliations:** a Pusan National University Hospital, Pusan National University School of Medicine, Department of Otorhinolaryngology and Biomedical Research Institute, Busan, South Korea; b Pusan National University Hospital, Pusan National University School of Medicine, Department of Pathology, Busan, South Korea

## Introduction

Metastasis to the orbit, which is uncommon due to the character of the orbital volume with relative stenosis, is estimated to account for 1–13% of all orbital tumors.^1^ Orbital metastasis is believed to occur in approximately 2–3% of patients with systemic cancer.^2^ The incidence of metastatic orbital tumors varies widely, according to geographical area and race; the most common primary cancers that metastasize to the orbit are breast, prostate, liver, and lung cancer.^1^, ^2^ Although nasopharyngeal carcinoma (NPC) involves the orbits through direct extension to the orbital apex, metastasis of NPC to the orbit has rarely been reported. This report describes two cases of intraorbital, extrabulbar metastases from NPC.

## Case reports

### Case 1

**Figure 1:**
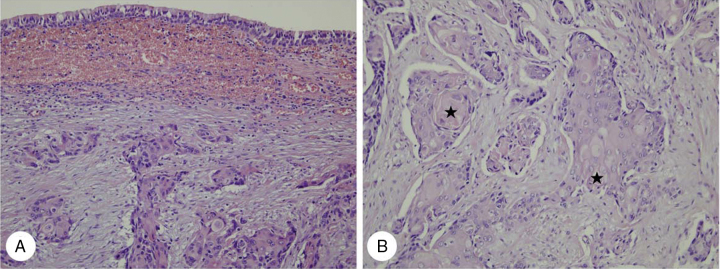
Histopathologic findings of case 1. (A) Infiltrating keratinizing carcinoma of the nasopharynx is noted beneath normal ciliated columnar pseudostratified epithelium (H&E, ×200). (B) Nasopharyngeal keratinizing carcinoma infiltrating orbital soft tissue exhibits distinct cytoplasmic keratin formation with pearl formation () (H&E, ×400).

A 52-year-old male with abrupt-onset hoarseness visited the authors’ clinic. His medical history was otherwise unremarkable. A 1 × 1 cm hard, fixed lymph node was palpable in the right level II area. Flexible fiberoptic laryngoscopy revealed right vocal cord paralysis. An ulcerative nasopharyngeal mass was observed by nasal endoscopy. Computed tomography (CT) of the neck revealed an enormous nasopharyngeal mass extending into the right oropharyngeal, masticator, carotid, prevertebral, and paravertebral space, with multiple bilateral cervical lymph nodes metastases. Transnasal endoscopic biopsy under local anesthesia and fine needle aspiration on the right level II lymph node were performed. Histopathologic examination revealed keratinizing squamous cell carcinoma (SCC) in both the nasopharyngeal mass and right cervical lymph node (Fig. 1A).

**Figure 2:**
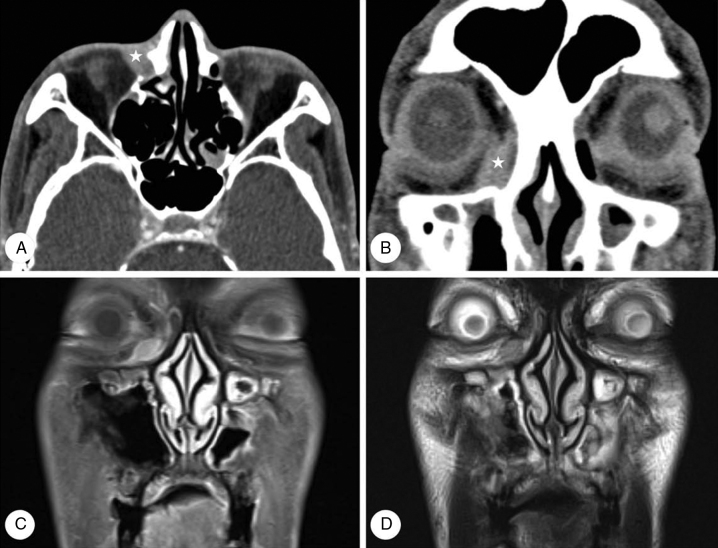
Computed tomography (CT) and magnetic resonance (MR) images of case 1. Axial (A) and coronal (B) CT images reveal a soft tissue mass with indistinct margins on the inferomedial side of the right orbit (). MR images reveal an oval-shaped orbital mass displaying mild enhancement on post-contrast T1 images (C) and intermediate signal intensity on T2 images (D).

The patient received docetaxel and cisplatin chemotherapy. However, chemotherapy was stopped after the first cycle due to neutropenic septic shock. Therefore, early radiotherapy of the nasopharynx and cervical lymph nodes (total dose = 70 Gy) was completed. After one month, mild swelling in the right medial canthal area was noted. Visual acuity, intraocular pressure, and ocular motility were within normal limits and there was no exophthalmos. Orbit CT revealed a newly developed 1.0 × 0.9 cm soft tissue mass with indistinct margins on the inferomedial side of the right orbit (Fig. 2A and B). Magnetic resonance (MR) images of the nasopharynx revealed an oval-shaped orbital mass displaying low signal intensity on T1-weighted images (T1WIs), intermediate signal intensity on T2WIs, and mild enhancement on gadolinium-T1WIs (Fig. 2C and D). This was followed by excisional biopsy that confirmed the diagnosis of keratinizing SCC (Fig. 1B). On positron emission tomography (PET)/CT scan, multiple areas of increased fluorodeoxyglucose (FDG) uptake were detected in the liver, diagnosed as distant liver metastases. Therefore, the patient received six cycles of palliative chemotherapy with TS-1 and cisplatin. Although primary NPC and orbital metastasis did not recur, the patient died 17 months after diagnosis of orbital metastasis due to liver failure.

### Case 2

**Figure 3:**
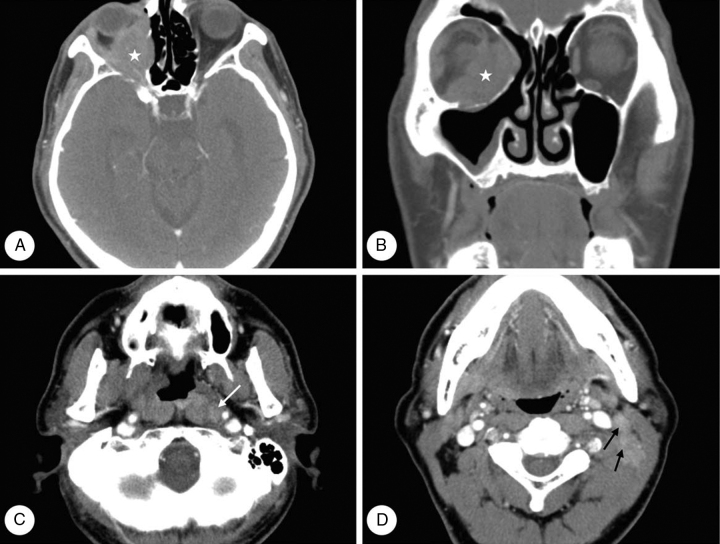
Computed tomography (CT) images of case 2. Axial (A, C, D) and coronal (B) images reveal a soft tissue mass () in the extraconal and intraconal space of the right orbit. Moreover, asymmetry of the left nasopharynx and metastatic lymph nodes is noted in the level II (black arrow) and retropharyngeal areas (white arrow) of the left neck.

**Figure 4:**
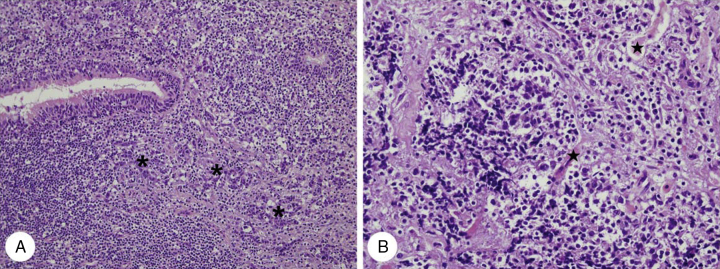
Histopathologic findings of case 2. (A) Infiltrating undifferentiated non-keratinizing carcinoma (*) of the nasopharynx is noted beneath normal ciliated columnar pseudostratified epithelium (H&E, ×200). (B) Nasopharyngeal undifferentiated carcinoma infiltrates orbital soft tissue and destroys extraocular striated muscles () (H&E, ×400).

A 67-year-old male presented with abrupt-onset ocular pain and blurred vision, which he had first noticed one month prior to presentation. He was seen by a neuro-ophthalmologist, who observed visual loss to no light perception on the right. The patient's medical history was otherwise unremarkable. Nasal endoscopy revealed a mild contour protrusion without mucosal ulceration or necrosis in the nasopharynx. Neck CT revealed a soft tissue mass in the extraconal and intraconal space of the right orbit. Moreover, asymmetry of the left nasopharynx and metastatic lymph nodes were present in the left level II, III, IV, and retropharyngeal areas (Fig. 3). On PET/CT scan, the main mass in the right orbit was of a hypermetabolic nature with a maximum standardized uptake value (maxSUV) of 3.5. Abnormal FDG uptake was present in multiple left neck lymph nodes, identical to CT findings. Transnasal endoscopic biopsy, of the right orbital mass and left nasopharyngeal mucosa, was performed under general anesthesia. Histopathologic examination of both specimens revealed undifferentiated carcinoma (Fig. 4).

The patient received two cycles of chemotherapy with docetaxel and cisplatin. However, he refused further treatment for economic reasons and died six months after orbital metastasis diagnosis.

## Discussion

NPC is a tumor that arises from epithelial cells covering the surface and lining of the nasopharynx.^3^ Three subtypes of NPC are recognized by the World Health Organization: keratinizing SCC, nonkeratinizing carcinoma, and undifferentiated carcinoma.^3^ Although NPC commonly metastasizes to cervical lymph nodes, orbital metastases are rare. The majority of orbital involvement cases involve direct invasion, typically via the pterygopalatine fossa and inferior orbital fissure, but occasionally through the ethmoid sinus and sphenoid sinus, into the apex, causing proptosis and muscle paralysis.^4^

Orbital metastatic tumors are characterized by rather abrupt onset of diplopia, blurred vision, and pain. A visible lump may also be present beneath the eyelid and progress is relatively rapid. Examination may disclose proptosis, displacement of the globe, blepharoptosis, and a visible or palpable mass.^1^, ^2^ The findings in the present cases accord with previous reports: orbital metastases became evident five months after NPC diagnosis in one patient and represented the first sign of NPC in the other.

The diagnosis of orbital metastasis should be suspected when a patient with a history of cancer exhibits the aforementioned symptoms. If the patient has no history of cancer, such findings should prompt a systemic survey to detect a primary neoplasm and other sites of metastasis. Although the ultimate diagnosis of orbital metastasis is rendered by biopsy or fine-needle aspiration, CT or MRI orbital imaging studies should also be preformed. CT is typically employed first because it provides better evaluation of bone. However, MRI usually provides the best resolution for orbital metastasis evaluation because the majority of orbital metastases principally affect orbital soft tissues. MRI typically reveals an inhomogeneous low signal mass on T1 images, increased signal intensity on T2 images, and a degree of enhancement with contrast agents.^5^ In the present cases, orbital metastasis from NPC was characterized by a diffuse or well-defined soft tissue mass, with low signal intensity on T1WIs and intermediate signal intensity on T2WIs with mild enhancement.

The main aim during treatment of orbital metastasis is to alleviate suffering and maintain visual function. Radiotherapy is the mainstay treatment for orbital metastasis from NPC, due to its sensitivity, but chemotherapy is also used in certain patients.^4^ If the tumor is well-circumscribed and amenable to complete removal, it should be treated by complete excisional biopsy.^1^ Prognosis is generally poor, because patients are typically at an advanced stage of disease. The overall mean survival time after orbital diagnosis is 15 months;^1^ one of this report's patients died after 17 months, the other after six months.

## Conclusion

Although NPC with orbital metastasis is rare, NPC can develop metastases in orbital and ocular regions. If NPC patients complain of ophthalmological symptoms such as local pain, impaired vision, eyelid swelling, or diplopia, it is important to consider orbital or ocular metastatic disease.

## Conflicts of interest

The authors declare no conflicts of interest.
